# No evidence for shared representations of task sets in joint task switching

**DOI:** 10.1007/s00426-016-0813-y

**Published:** 2016-10-15

**Authors:** Motonori Yamaguchi, Helen J. Wall, Bernhard Hommel

**Affiliations:** 1 Department of Psychology, Edge Hill University, St Helens Road, Ormskirk, Lancashire L39 4QP UK; 2 Institute of Psychology, Leiden University, Leiden, The Netherlands

## Abstract

It has been suggested that actors co-represent a shared task context when they perform a task in a joint fashion. The present study examined the possibility of co-representation in *joint task switching*, in which two actors shared two tasks that switched randomly across trials. Experiment 1 showed that when an actor performed the tasks individually, switch costs were obtained if the actors responded on the previous trial (go trial), but not if they did not respond (no-go trial). When two actors performed the tasks jointly, switch costs were obtained if the actor responded on the previous trial (actor-repeat trials) but not if the co-actor responded (actor-switch trials). In Experiment 2, a single actor performed both tasks of the joint condition to test whether the findings of Experiment 1 were due to the use of different response sets by the two actors. Switch costs were obtained for both repetitions and alternations of the response set, which rules out this possibility. Taken together, our findings provided little support for the idea that actors co-represent the task sets of their co-actors.

## Introduction

There are numerous occasions in everyday activities for which two or more individuals need to perform a task cooperatively to achieve a common goal. In such situations, the labor required to perform the shared task must be divided between co-acting individuals. For instance, a person may drive a car while another navigates the driver in an unfamiliar neighborhood. The driver is concerned with the operations of the vehicle and the traffic condition, whereas the navigator is concerned with the current location of the vehicle and the selection of the correct route to the destination. Given that each actor possesses only an incomplete picture of the whole task context, how can they achieve a common goal that requires information from both actors? Traditional approaches suggest that the actions of one actor can become stimuli to trigger the actions of the other, and vice versa, until the shared goal is achieved. However, a recent approach has suggested a more far-reaching possibility that co-actors do not only represent the shared goal, but also co-represent the entire task that includes both the actor’s own context and the co-actor’s context (Sebanz, Knoblich, & Prinz, 2003; Knoblich, Butterfill, & Sebanz, 2011). By doing so, each co-actor would represent a given task in the same fashion, which implies that each co-actor would represent the task-related stimulus–response mappings not only for his or her own part of the task, but also for the part of the co-acting other’s.

Previous research has provided evidence for the assumption that jointly performing a task leads to the representation of at least some aspects of a co-actor’s contributions to the task. The most systematic findings pertaining to this issue have been obtained by means of the *joint Simon task* (Sebanz et al., 2003). In a standard, individual Simon task, participants press a left or right key in response to non-spatial features of a stimulus that is presented randomly to the left or right of some reference point (e.g., the fixation mark on the monitor). Even though stimulus location is irrelevant to selecting the correct responses, responses are faster and more accurate if the stimulus location coincides with the response location than if it does not, which is termed the *Simon effect* (Lu & Proctor, 1995; Yamaguchi & Proctor, 2012). In the joint version of the task, the two responses are divided among two co-acting participants, such that one actor responds to one of the relevant stimulus features (e.g., red stimuli) and the other actor responds to the other stimulus features (green stimuli). Note that this renders the task essentially a go/no-go task, which does not yield a Simon effect in the absence of a co-actor (Hommel, 1996). In the presence of a co-actor who operates the other key, a reliable Simon effect is observed (Sebanz et al., 2003), which is thus termed the *joint Simon effect*. Given that the Simon effect is attributed to response-selection processes (Lu & Proctor, 1995; Hommel, 2011), the joint Simon effect demonstrates that participants take into consideration the active contributions of their co-actor when selecting their responses.

Research has begun to determine in more detail which aspects of the co-actor’s contributions to the task are considered in the process of response selection. Earlier approaches assumed that the impact of the co-actor’s contributions on response selection is automatic (Sebanz et al., 2003; Sebanz, Knoblich, & Prinz, 2005) and only occurs with human co-actors (e.g., Tsai, Kuo, Hung & Tzeng, 2008), which has been taken to demonstrate the “social nature of perception and action” (Knoblich & Sebanz, 2006). More recent studies have shown that the joint Simon effect is sensitive to the relationship between the two co-actors, so that its presence or size depends on whether this relationship is positive or negative (Hommel, Colzato & van den Wildenberg, 2009), competitive or cooperative (Ruys & Aarts, 2010), or more or less empathic (Ford & Aberdein, 2015), and whether the co-actors are perceived to belong to the same social group (Aquino et al., 2015; Constantini & Ferri, 2013; Iani, Anelli, Nicoletti, Arcuri, & Rubichi, 2011; McClung, Jentzsch & Reicher, 2013). These observations suggest that the joint Simon effect relies on particular social factors, although they do not necessarily support a strong claim that all perception–action processes are inevitably social in nature (see Dolk et al., 2014, for a review). Moreover, a reliable joint Simon effect is obtained not only with human or anthropomorphized non-human co-actors (Müller et al., 2011), but also with a salient inanimate object such as a Japanese waving cat or a ticking metronome that is presented in the place of a co-actor (Dolk, Hommal, Colzato, Prinz & Liepelt, 2011).

Although these findings do not warrant a strong claim that co-actors *fully* share their entire task experience, the findings would arguably allow for a more parsimonious interpretation of task sharing that an actor represents the actions of, or even the stimulus–response relationships followed by, a co-actor. It is possible that participants represent not only the stimuli that they need to respond to (which in some sense already implies some knowledge about the stimuli they need to ignore) and the responses that they need to carry out, but also the activities that their co-actor carries out. This might be because it allows the better monitoring of turn taking in a joint task (Liefooghe, 2016; Wenke et al., 2011), or because the presence of another event, such as the co-actor’s activities, reintroduces an event representation against which the instructed event (one’s own action) needs to be selected (Dolk et al., 2014). It is even possible that people represent the associations between stimuli and responses for both one’s own and the other’s actions, as this would allow one to predict actions from stimuli. Such associations are commonly assumed to constitute the basis of task sets, which would suggest that people do represent at least the basic ingredients of the task set of a co-actor (Knoblich et al., 2011).

The present study aimed to disentangle these two possibilities by means of a *joint task*-*switching setting*. In regular, individual task-switching settings, participants alternate between two or more tasks that differ with respect to the assignment of responses to stimuli. Responses are commonly faster if the task on the current trial is the same as the preceding trial (repeat trial) than if it is different (switch trial)—the so-called switch cost (for reviews, see Kiesel et al., 2010; Vandierendonck, Liefooghe, & Verbruggen, 2010). Switch costs are likely to reflect a larger number of processes, including the extra time needed to reconfigure the task set on switch trials (Meiran, 1996), interference from no longer relevant but still lingering previous task sets (Allport, Styles, & Hsieh, 1994), priming in case of a repeated task cue (Logan & Bundesen, 2003), and the residual switch costs that remain even after a long preparation time (Rogers & Monsell, 1995). Consider how switch costs might be affected by having tasks being shared by two co-actors. The most obvious prediction from a shared task set account (Knoblich et al., 2011) would be that switch costs should be the same irrespective of whether the previous trial was carried out by the participant or a co-actor. Previous studies do not provide unequivocal evidence for this possibility.

To date, three studies have looked into joint task switching (Dudarev & Hassin, 2016; Liefooghe, 2016; Wenke et al., 2011). Two of these studies had two actors perform two different tasks with the relevant actor being cued randomly from trial to trial (Dudarev & Hassin, 2016; Liefooghe, 2016). Whereas this design implies confounding between task switching and turn taking (i.e., switching the actor implied switching the task, and vice versa), the idea was that switch trials should produce worse performance than repeat trials only if the participant actively represents the co-actor’s task. Thus, the presence of switch costs after co-actor’s trials would indicate shared task representations. Dudarev and Hassin (2016) obtained significant switch costs in such a joint condition, but not in a control condition for which individual participants carried out the same go/no-go task without a co-actor. The researchers also found no switch costs in another control condition for which both actors worked on the same task. The results were taken to argue against the possibility that turn taking itself could have been responsible for the measured switch costs. Unfortunately, however, the same task versus different task manipulation was carried out between groups, which might have affected the representation of the other agent. A co-actor who performs the same task as oneself may be perceived as more similar to oneself (Hommel et al., 2009) than a co-actor who performs a different task. Perceived self–other similarity has been demonstrated to facilitate “feature migration” between self and other (Ma, Sellaro, Lippelt & Hommel, 2016), in the sense that features that actually relate to the other are perceived as part of oneself. Thus, it may be that switching is more demanding between actors that are perceived to be more different, producing switch costs in the different task conditions but not in the same task condition. This explanation would not require the assumption of task sharing. It may also be that participants monitor the stimulus–response contingencies of the co-actor’s trials (Liefooghe, 2016), which would necessarily lead to more violations of the rules the participant is storing for his or her own performance if the co-actor’s task is different, especially when the same stimuli are mapped to different responses for two co-actors (as was the case in Dudarev and Hassin’s study). Such violations might have increased the dissimilarities between the co-actors, making it more difficult to perform the task after the co-actor’s trial.

A very similar study was carried out by Liefooghe (2016), who also had co-actors carry out different tasks. The study aimed to separate three different components of switch costs: (a) the efficiency of task preparation, as measured by the reduction of switch costs if more preparation time is available; (b) the interference from the previous task set, as measured by the reduction of switch costs if the interval between the previous response and the next task cue increases; and (c) the residual switch costs that remain after the longest preparation time. Interestingly, neither task preparation nor interference from the previous task was sensitive to task/actor switches, which according to the author rules out that participants truly represented the co-actor’s task set. In contrast, residual switch costs were increased with task/actor switches, an effect that Liefooghe attributed to the requirement to identify the relevant actor in this condition. This interpretation would also account for the findings of Dudarev and Hassin (2016), as would the aforementioned possibility that exposure to different stimulus–response contingencies increases cognitive conflict.

Whereas the studies of Dudarev and Hassin (2016) and Liefooghe (2016) can be taken to investigate the effects of actor switching, Wenke et al. (2011, Experiment 2) discuss an unpublished study that assessed the impact of task switching more directly. This study sought to separate the effects of actor switching in task switching by having two actors perform the same two tasks (e.g., color discrimination and shape discrimination). A task and an actor were randomly cued on each trial, so that only one actor performed on a given trial, just as in the studies of Dudarev and Hassin (2016) and Liefooghe (2016). This joint go/no-go (i.e., actor no switch and switch) condition was compared to an individual go/no-go condition, in which a single actor performed the same task with the actor cue serving a go/no-go cue (i.e., ‘respond’ vs. ‘not respond’). There were switch costs after go trials and no switch costs after no-go trials in the individual condition, which replicated a standard observation (e.g., Schuch & Koch, 2003). The crucial question was whether the same pattern could be observed in the joint condition. If the actor repeats (i.e., in go trials), one would expect standard switch costs, as the participant would need to reconfigure his or her own cognitive system. More diagnostic data came from the actor-switch trials, as these followed no-go trials. If participants represent the co-actor’s task set just like their own (Knoblich et al., 2011), one would expect switch costs of the same size as in trials following go trials, so that switching costs should be independent from actor switch. If they do not represent the co-actor’s task set, however, one would expect no switch costs just as in the individual condition. Wenke et al. (2011) report that the same pattern was found in individual and joint conditions, with significant switch costs for actor repetitions but not for actor switches.

While this would arguably be rather strong evidence against the shared representation of task sets, the respective study has not yet been published and the brief description presented in Wenke et al.’s (2011) review article does not allow for strong and far-reaching claims. To test whether the necessary evidence could be provided, Experiment 1 of the present study conceptually replicated and extended the experiments discussed by Wenke and colleagues, which allowed us to avoid the problems associated with the two actor-switching studies of Dudarev and Hassin (2016) and Liefooghe (2016). Note that in Experiment 1, actor switching was de-confounded from task switching, but was still confounded with switching of response set because two co-actors used different sets of response keys. Experiment 2 examined the effect of switching response sets on task switch costs by having a single participant perform both tasks of the joint condition using the two response sets that were assigned to two co-actors in Experiment 1. If the same task set is used to perform the same task with different response sets, switch costs should be obtained regardless of whether the response set is switched. Such outcomes would reinforce the interpretation of Experiment 1, especially if switch costs are found to depend on actor switching.

## Experiment 1

In Experiment 1, pairs of individuals performed a cued task-switching paradigm, in which a task cue unpredictably signaled one of two tasks (color or shape task) on each trial. The actors used two different sets of two response keys each to respond to the target stimuli. In the *joint condition*, one of the actors was also cued at target onset, and only the cued actor responded while the other did not perform. In the *individual condition*, the procedure was identical, except that one of the actors did not perform throughout an entire block, so no one responded on trials for which the active actor was not cued. It was expected that in the individual condition, switch costs should be obtained on trials that followed go trials, but not on trials that followed no-go trials (Schuch & Koch, 2003). If task sets are co-represented by the co-acting participants in the joint condition, there should be switch costs on trials that followed the co-actor’s trial as well as on trials that followed the actor’s own trial. If not, the outcome should be comparable for joint and individual conditions, with switch costs being present in trials following go trials but not in trials following no-go trials.

Note that Wenke et al.’s conclusion relied on the null effect including only 16 participants in each of the two versions of the experiment. We used a larger sample size (*N* = 56) to increase the statistical power^1^ so that small effects could be detected. Moreover, none of the previous studies included multiple task cues for each task, so the contribution of cue priming was not dissociated from that of task switching (Logan & Bundesen, 2003). By including multiple task cues, we could examine whether the actors pay attention to some aspects of the co-actor’s task context if not to the entire context, as recently suggested in a different joint task setting (Janczyk, Welsh, & Dolk, 2016). Thus, we included three types of transitions, cue-repeat trial (both the task cue and the task repeated), cue-switch trial (the task cue switched, but the task repeated), and task-switch trial (both the task cue and the task switched). Each transition occurred in one-third of the trials. The difference between cue-repeat and cue-switch trials reflected a cue-switch cost, and the difference between cue-switch and task-switch trials reflected a task-switch cost. We expected no task-switch cost on trials following no-go trials in the individual and joint conditions, as in Wenke et al.’s report. It was still possible to obtain cue-switch costs in the joint condition, because the task cue appeared before the actor cue, so both actors would have to encode the task cue on every trial. If so, the encoded task cue would remain in short-term memory and facilitate cue encoding on the next trial (Logan & Bundesen, 2003), facilitating responding when the same task cue repeats.

## Method

### Participants

Fifty-six undergraduate students at Edge Hill University participated in the present study (49 females, 7 males; mean age 18.79, SD 1.41, range 18–24). They were recruited from an introductory psychology module and received experimental credits toward the module or paid £3 for participation. All reported having normal color vision and normal or corrected-to-normal visual acuity. They were naïve as to the purpose of the experiment.

### Apparatus and stimuli

The apparatus consisted of a personal computer and a 23-in widescreen monitor. Stimuli were green and red squares (4.8 cm in side) and diamonds (the squares tilted 45°), which appeared at the center of the screen. The task cues were “COLOUR” and “HUE” for the color task, and “SHAPE” and “FORM” for the shape task. The task cues were presented in the Courier New font at 36-pt. They appeared 6.8 cm above the screen center. The actor cue was the letter “A” (to indicate the actor on the left) and “B” (to indicate the actor on the right). The cue was superimposed on diamonds and squares, in the Arial font at 40-pt in white; as the background was also white, it appears as if there was a letter-shaped hole in the stimulus. Responses were registered by pressing keys on a QWERTY desktop keyboard.

### Procedure

The experiment was conducted in two computer labs with 24 seats arranged in four rows of six computers each. The distance between two adjacent computers was about 160 cm. There were at most three pairs in each row; each pair of participants was seated in front of a computer monitor and at every other computer to avoid cluttering between pairs. Participants from different seminar groups were assigned to pairs randomly by the experimenter. Each pair of participant read on-screen instructions, which emphasized both the speed and accuracy of the response. Participants who sat on the left side placed their left and right index fingers on the ‘z’ and ‘c’ keys, respectively; those who sat on the right side placed their left and right index fingers on the ‘1’ and ‘3’ keys on the numerical keypad on the right side of the keyboard. For both participants, the ‘z’ and ‘1’ keys were called the left response, and the ‘c’ and ‘3’ keys were the right response. Each participant was instructed to press the left key for one color and the right key for the other color for the color task, and the left key or one shape and the right key or the other shape for the shape task; the mappings between the keys and the colors and shapes were randomly determined for each pair.

Each participant performed two *joint blocks*, for which one participant responded to a subset of stimuli and another to another subset of stimuli, and one *individual block*, for which one participant responded to stimuli and another participant remained silent. Thus, there were two joint blocks and two individual blocks for each pair. Each block consisted of 120 test trials, and there was a block of 16 practice trials before the first joint blocks and before each of the two individual blocks (one for each actor). For some pairs, two joint blocks were administered first and then two individual blocks; for other pairs, two individual blocks were administered first and then two joint blocks. The order of the joint and individual blocks was determined randomly for each pair. Within the individual blocks, the order of the actor performing the block was also determined randomly.

In the joint block, each trial started with a task cue that stayed on the screen for 450 ms, followed by a 50-ms blank screen. The imperative stimulus (colored square or diamond) appeared for 2000 ms or until a response was made, along with the actor cue that was superimposed on the imperative stimulus. If the correct response was made, a blank display replaced the stimulus and lasted for 1000 ms; otherwise, an error message was presented for 1000 ms. The message was “Error!” for an incorrect response and “Faster!” for no response. If a wrong actor responded, the message was “Not your turn!” The next trial started with another task cue. Response time (RT) was measured as the interval between the onset of the imperative stimulus and a depression of a response key.

The individual block was essentially the same, but participants were required to respond only when the actor cue indicated their trials (go trials) but withhold responding when the actor cue indicated their co-actor’s trials (no-go trials). If no response was made on a go trial, the error message was “Respond!” If a response was made on a no-go trial, the error message was “Don’t respond!” There was a 2000-ms window to respond on each trial.

### Design

The experiment involved two conditions, joint and individual conditions, which defined the factor Task Condition. Both conditions consisted of three types of task sequences (cue repeat, cue switch, and task switch), which defined the factor Task Sequence. Cue repeat referred to the condition for which the same task cue occurred on two successive trials (e.g., “SHAPE” on trial N, and “SHAPE” again on trial *N* + 1); cue switch referred to the condition for which the two different task cues assigned to the same task occurred on two successive trials (e.g., “SHAPE” on trial N, and “FORM” on trial *N* + 1); and task switch referred to the condition for which two different task cues assigned to different tasks occurred on two successive trials (e.g., “SHAPE” on trial N, and “HUE” on trial *N* + 1). Trials were determined randomly on each trial, so that there was an equal probability of 33 % for each sequence type. In the individual condition, previous trials could be go or no-go trials; in the joint condition, previous trials could be performed by the same actor as the current trial (*actor repeat*) or by a different actor (*actor switch*). The factor Previous Trial was defined as to whether the same actor responded to stimuli on the previous trial (go trials in the individual condition and actor-repeat trials in the joint condition) or did not (no-go trials in the individual condition and actor-switch trials in the joint condition). Previous Trial and Task Sequence were manipulated orthogonally.

## Results

**Fig. 1:**
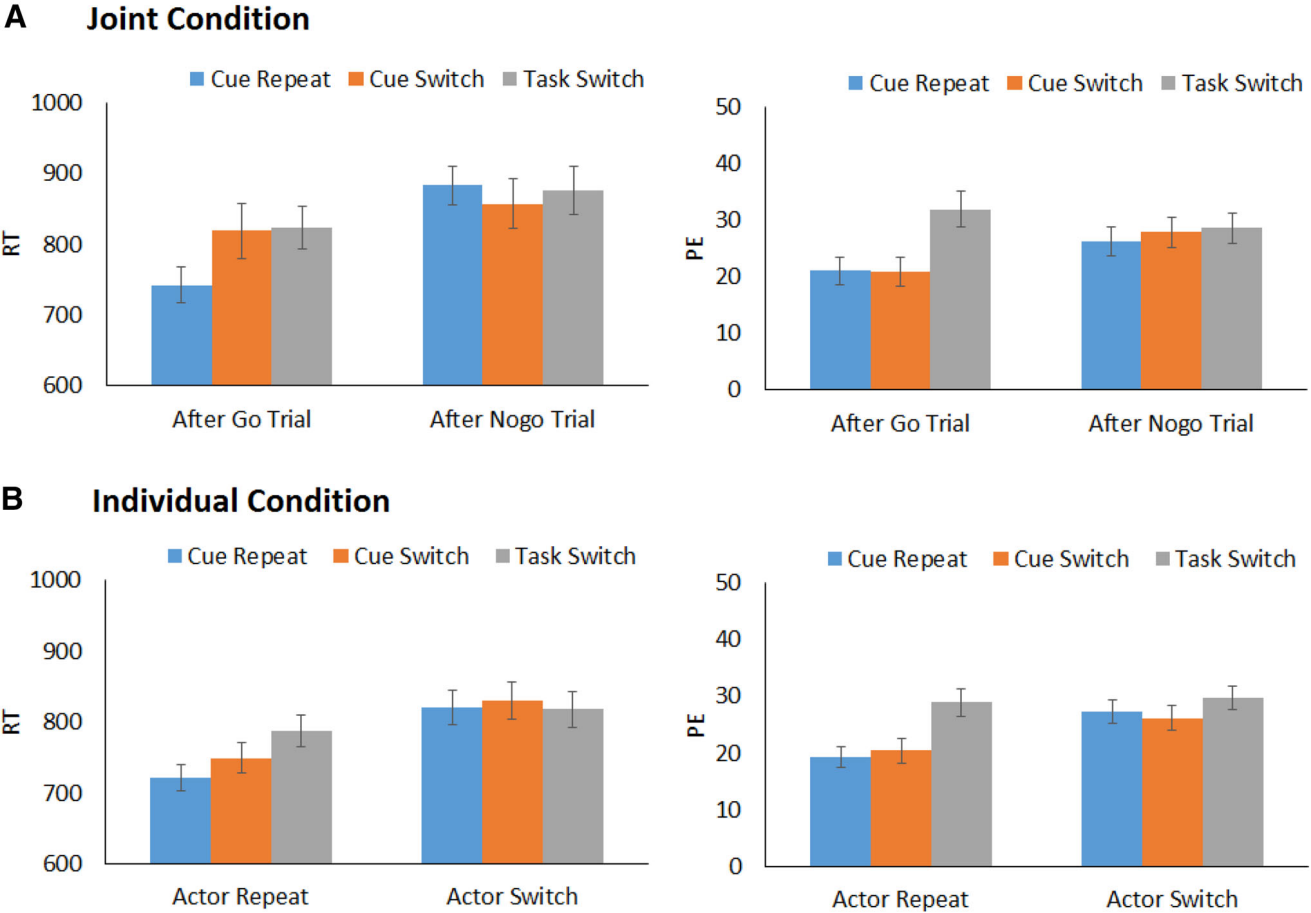
Mean response times (RT) and percentages of error trials (PE) for the joint condition (**a**) and the individual condition (**b**) as a function of task sequence and the previous trial in Experiment 1 (*error bars* represent one standard error of the mean)

Trials were excluded from analyses if RT was less than 200 ms, if no response was made within the 2000-ms window, or if a wrong actor responded (4.44 % of all trials). Among the remaining trials, the overall error rate was (25.31 %), which is higher than typical task-switching experiments with single actors. This is reasonable given the complexity of the task. Also, participants did not receive extensive practice with the task before the test trials, which might have contributed to increasing the overall error rates. Due to the high error rates, trials that followed an error trial were not excluded to retain as many trials as possible (the data were also analyzed after excluding trials following an error trial, but the results are consistent with those reported below). One female participant was excluded from the analysis due to an empty case in one of the conditions. Mean RTs and percentages of error trials (PE) were computed for each participant and submitted to 2 (Task Condition: joint vs. individual) × 3 (Task Sequence: task switch vs. cue switch vs. cue repeat) × 2 (Previous Trial) ANOVAs. All factors were within-subject variables. The ANOVA results are summarized in Table 1. RT and PE are shown in Fig. 1.

**Table 1:** ANOVA results in Experiment 1

Factors	*df*	MSE	*F*	*p*	*η*_p_^2^
*Response time*					
Task condition (TC)	**1, 54**	**80,193.05**	**4.29**	**0.043**	**0.074**
Previous trial (PT)	**1, 54**	**10,378.33**	**43.8**	**<0.001**	**0.448**
Task sequence (TS)	**2, 108**	**12,456.51**	**5.28**	**0.006**	**0.089**
TC × PT	1, 54	9779.98	<1	0.611	0.005
TC × TS	2, 108	12,255.84	<1	0.940	0.001
PT × TS	**2, 108**	**8840.93**	**10.58**	**<0.001**	**0.164**
TC × PT × TS	2, 108	11,723.51	2.3	0.105	0.041
*Percentage of error trials*					
TC	1, 54	548.72	<1	0.679	0.003
PT	**1, 54**	**109.30**	**22.60**	**<0.001**	**0.295**
TS	**2, 108**	**166.57**	**16.47**	**<0.001**	**0.234**
TC × PT	1, 54	145.18	1.05	0.310	0.019
TC × TS	2, 108	117.26	<1	0.918	0.002
PT × TS	**2, 108**	**112.29**	**9.89**	**<0.001**	**0.155**
TC × PT × TS	2, 108	148.02	<1	0.504	0.013

Bold represents a significant effect

### Mean response times

Responses were generally faster for the joint condition (*M* = 788 ms) than for the individual condition (*M* = 833 ms), as indicated by a significant main effect of Task Condition. A significant main effect of Previous Trial revealed that responses were also faster if the previous trial was a go trial (*M* = 795 ms) or the actor’s own trial (*M* = 753 ms) than if the previous trial was a no-go trial (*M* = 872 ms) or the co-actor’s trial (*M* = 823 ms). A main effect of Task Sequence was also significant, and the factor interacted with Previous Trial. Post hoc tests (Bonferroni adjusted^2^) compared RTs for cue-repeat, cue-switch, and task-switch trials to clarify the interaction. When the same actor responded on the previous trial, RT was shorter for cue-repeat trials (*M* = 732 ms) than for cue-switch trials (*M* = 784 ms, *p* = 0.004) and for task-switch trials (*M* = 806 ms, *p* < 0.001), whereas the latter two did not differ (*p* = 0.390). However, when the same actor did not respond on the previous trial (i.e., when the previous trial was no-go in the individual condition or when it was the co-actor’s trial in the joint condition), no switch costs emerged (*M*s = 852 ms for cue repeat, 843 ms for cue switch, and 847 ms for task switch; all *ps* = 1).

Although the three-way interaction among Previous Trial, Task Sequence, and Task Condition was far from significant, we also compared the three trial types in terms of previous trial separately for the individual and joint conditions for clarity. In the individual condition, when the previous trial was a go trial, RT was shorter for cue-repeat trials (*M* = 742 ms) than for cue-switch trials (*M* = 819 ms; *p* = 0.038) and for task-switch trials (*M* = 823 ms; *p* = 0.002), whereas the latter did not differ (*p* = 1). When the previous trial was a no-go trial, there were no differences among the three task sequences (*M*s = 883 ms, 857, ms, 876 ms, for cue-repeat, cue-switch, and task-switch trials; all *p*s = 1). In the joint condition, when the previous trial was the actor’s own trial, RT was shorter for cue-repeat trials (*M* = 722 ms) than task-switch trials (*M* = 788 ms; *p* < 0.001) and tended to be shorter for cue-repeat trials than for cue-switch trials (*M* = 749 ms; *p* = 0.073) and for cue-switch trials than for task-switch trials (*p* = 0.071). Most importantly, when the previous trial was the co-actor’s trial, RT did not differ for these trials (*M*s = 821, 830, and 818 ms, for cue-repeat, cue-switch, and task-switch trials, respectively; all *p*s = 1).

### Percentages of error trials

The PE results were consistent with the RT results, except that a main effect of Task Condition was not significant. A significant main effect of Previous Trial revealed that PE was smaller when the same actor responded on the previous trial (*M* = 23.73 %) than when the actor did not respond (*M* = 27.60 %). Task Sequence produced a main effect, and it also interacted with Previous Trial. When the same actor responded on the previous trial, task switch produced a larger PE (*M* = 30.40 %, *p* < 0.001) than did cue repeat (*M* = 20.16 %, *p* < 0.001) or cue switch (*M* = 20.64 %), whereas the latter two did not differ (*p* = 1). When the actor did not respond on the previous trial, there were no differences among the three sequences (*M*s = 26.72 % for cue repeat, 27.00 % for cue switch, and 29.08 % for task switch; all *p*s > 0.4). As in RT, Task Condition did not modulate these outcomes.

## Discussion

As expected, switch costs were obtained in the individual condition when the preceding trial was a go trial, but not when it was a no-go trial. Importantly, switch costs were also obtained in the joint condition when the preceding trial was the actor’s own trial (actor repeat), but not when it was the co-actor’s trial (actor switch). Note that go/no-go signals appeared with the target after the presentation of a task cue, so task preparation would have occurred on each trial, but response selection might have been restricted to go trials (Schuch & Koch, 2003). This is consistent with the assumption that the actors in the present experiment did not select a response on their co-actors’ trials as if these trials were their own (Wenke et al., 2011), which is not consistent with the idea that the task set of the co-actor was co-represented (Knoblich et al., 2011). Furthermore, the inclusion of multiple task cues for each task did not show any evidence that task cue priming facilitated responding after no-go or the co-actor’s trials. This is an interesting outcome. Cue repetition is thought to allow the actors to retain the previous task cue in short-term memory and facilitate cue encoding on the current trial (Logan & Bundesen, 2003). The task cue appeared before the actor cue in the present experiment, so the actors would need to encode the task cue on every trial. The lack of cue switch costs after no-go trials indicates that the encoded task cue was discarded from short-term memory during the co-actor’s trial. One possibility is that the representation of the given task cue was bound to either one’s own no-go reaction (cf., Kühn & Brass, 2010) or to the other agent. Repeating a cue could then have retrieved this (now misleading) binding, which would be expected to create conflict (Hommel, 2004) and might have counteracted possible priming benefits. In any case, this effect does not support co-representation of co-actor’s task sets either.

It is also interesting to note that participants made relatively fewer errors to interpret the actor cue (<5 %) and the majority of errors was due to misapplications of wrong stimulus–response mappings (~25 %). These outcomes may reflect a hierarchical structure of cognitive processes, whereby participants first determined whose turn it was and subsequently selected an appropriate response to the target.

## Experiment 2

Although the outcome of Experiment 1 is consistent with those of the study discussed in Wenke et al.’s (2011) review, one may wonder how the obtained switch costs compare to standard switch costs obtained when a single actor performs the same task condition without a co-actor. We call this a full-task condition, following Liefooghe (2016) who also compared actor switching with standard individual task switching. Testing a full-task condition is important, because a lack of switch costs in the joint condition of Experiment 1 can be taken as evidence against a strong shared task set, only if switch costs could be obtained in the full-task condition. This is because of the possibility that assigning different tasks to different hands or response sets facilitates the cognitive separation and discrimination between the two tasks, which in turn might reduce or eliminate task switch costs (Jersild, 1927). Only if this possibility can be excluded, can we confidently conclude that the outcome of Experiment 1 provides unequivocal evidence against task set sharing in this task setting. Therefore, in Experiment 2, we went on to test whether switch costs are obtained in a full-task condition: a single actor used the two response sets that were distributed between two actors in the joint condition of Experiment 1, with one hand operating one response set and the other hand operating the other response set. The actor cue used in Experiment 1 was used to cue a response set (i.e., hand) to be used on a given trial. Because a single actor represented both task contexts, we expected that switch costs should be obtained regardless of whether the response set was switched across successive trials.

## Method

### Participants

Twenty-six participants were recruited from the Edge Hill University community (19 females, 7 males; mean age 21.73, SD 6.57, range 18–50). They received experimental credits toward their psychology modules or paid £3 for participation. All reported having normal color vision and normal or corrected-to-normal visual acuity. They were naïve as to the purpose of the experiment.

### Apparatus, stimuli, and procedure

The apparatus and stimuli were identical with those used in Experiment 1. The only difference was that participants performed all trials alone without a co-actor. The experiment was also conducted individually in a smaller experimental room. Participants placed their left middle and index fingers on the ‘z’ and ‘c’ keys and their right index and middle fingers on the ‘1’ and ‘3’ keys on the numeric keypad, respectively. The letter that appeared within the target stimulus cued which hand to use, such that “A” indicated the left hand and “B” indicated the right hand.

Each participant performed two blocks of the go/no-go condition and two blocks of the full-task blocks. The go/no-go condition was exactly the same as the individual condition in Experiment 1, except that there was no co-actor sitting next to them; participants always used the left hand on one block and the right hand on the other block. The full-task condition was the same as the joint condition in Experiment 1, except that the actor cue now cued the hand to be used on each trial (“A” indicated the left hand, and “B” indicated the right hand). Two blocks were identical for the full-task condition. The present experiment followed closely the procedure of Experiment 1 in other respects.

### Design

The experiment involved two conditions, full-task and go/no-go conditions, which were analogous to the joint and individual conditions in Experiment 1, respectively, and defined the factor Task Condition. Both conditions consisted of three types of task sequences (cue repeat, cue switch, and task switch), which defined the factor Task Sequence. In the go/no-go condition, previous trials could be a go or no-go trial; in the full-task condition, previous trials could be performed with the same hand as the current trial (*hand repeat*) or by a different hand (*hand switch*). The factor Previous Trial was defined as to whether participants used the same hand to respond to stimuli on the previous trial (go trials in the go/no-go condition and hand repeat trials in the full-task condition) or they did not (no-go trials in the go/no-go condition and hand switch trials in the full-task condition).

## Results

**Fig. 2:**
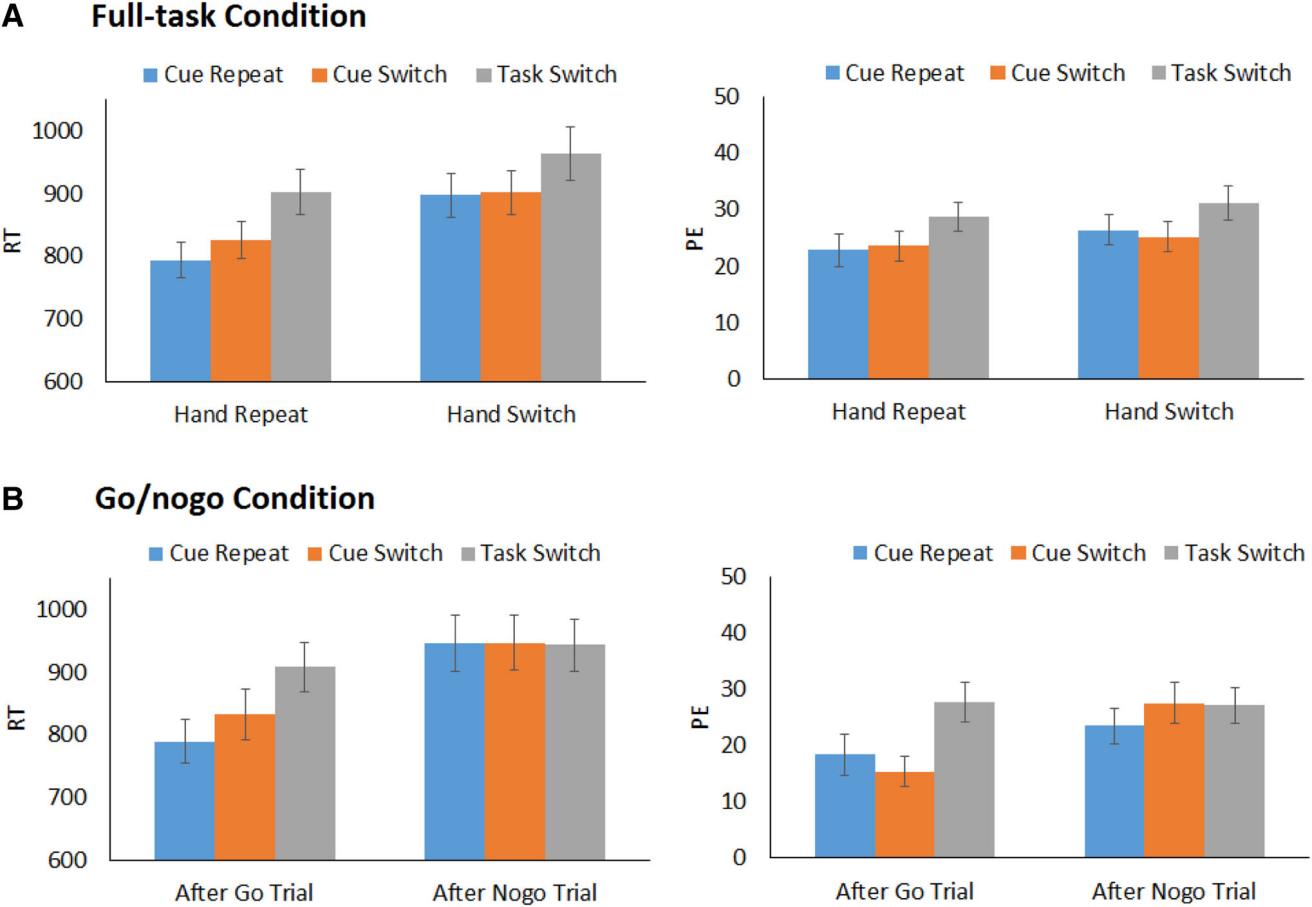
Mean response times (RT) and percentages of error trials (PE) for the full-task condition (**a**) and the go/no-go condition (**b**) as a function of task sequence and the previous trial in Experiment 2 (*error bars* represent one standard errors of the mean)

Trials were excluded in the same manner as in Experiment 1 (4.18 % of all trials). As in Experiment 1, the overall error rate was high (33.55 %). Trials that followed an error trial were excluded in the analysis, but the results were consistent when those trials were included. RT and PE were computed for each participant and submitted to 2 (Task Condition: full-task vs. go/no-go) × 3 (Task Sequence: task switch vs. cue switch vs. cue repeat) × 2 (Previous Trial) ANOVAs. The ANOVA results are summarized in Table 2. RT and PE are shown in Fig. 2.

**Table 2:** ANOVA results in Experiment 2

Factors	*df*	MSE	*F*	*p*	*η*_*p*_^2^
*Response time*					
Task condition (TC)	1, 25	81,374.85	<1	0.676	0.007
Previous trial (PT)	**1, 25**	**14,310.02**	**45.39**	**<0.001**	**0.645**
Task sequence (TS)	**2, 50**	**6,751.92**	**21.72**	**<0.001**	**0.465**
TC × PT	1, 25	7,016.36	1.35	0.257	0.051
TC × TS	2, 50	3,633.36	2.35	0.106	0.086
PT × TS	**2, 50**	**4,929.93**	**8.97**	**<0.001**	**0.264**
TC × PT × TS	**2, 50**	**3,397.35**	**3.68**	**0.032**	**0.128**
*Percentage of error trials*					
TC	1, 25	420.34	1.80	0.192	0.067
PT	**1, 25**	**150.26**	**8.52**	**0.007**	**0.254**
TS	**2, 50**	**94.58**	**12.51**	**<0.001**	**0.333**
TC × PT	1, 25	100.21	1.84	0.187	0.069
TC × TS	2, 50	115.36	<1	0.925	0.003
PT × TS	**2, 50**	**67.34**	**3.55**	**0.036**	**0.124**
TC × PT × TS	**2, 50**	**82.47**	**3.73**	**0.031**	**0.130**

Bold represents a significant effect

### Mean response times

The significant main effect of Previous Trial revealed that responses were faster when participants used the same response set on the previous trial (*M*s = 843 ms and 841 ms for the go/no-go and full-task conditions, respectively) than when they did not (*M*s = 945 ms and 921 ms for the go/no-go and full-task conditions, respectively). Responses also depended on Task Sequence, but this effect was modulated by Previous Trial and Task Condition. To clarify the three-way interaction, the effect of Task Sequence was analyzed separately for the full-task condition and the go/no-go condition. Multiple comparisons were Bonferroni corrected.

For the full-task condition, RT was slower for task-switch trials (*M* = 902 ms) than for cue-switch trials (*M* = 827 ms; *p* = 0.001) or for cue-repeat trials (*M* = 793 ms; *p* < 0.001) when the previous trial was performed by the same hand, analogous to actor-repeat trials in the joint condition of Experiment 1; there were no differences between cue-repeat and cue-switch trials (*p* = 0.109). When the previous trial was performed by the different hand, RT was still longer for task-switch trials (*M* = 964 ms) than for cue-switch trials (*M* = 901 ms; *p* = 0.003) or cue-repeat trials (*M* = 897 ms; *p* = 0.004); there were no differences between cue-repeat and cue-switch trials (*p* = 1).

For the go/no-go condition, RT was longer for task-switch trials (*M* = 909 ms) than for cue-switch trials (*M* = 832 ms; *p* = 0.001) or cue-repeat trials (*M* = 789 ms; *p* < 0.001) when the previous trial was a go trial; there was no difference between the latter two (*p* = 0.225). When the previous trial was a no-go trial, no difference emerged among task switch (*M* = 943 ms), cue switch (*M* = 947 ms), and cue repeat (*M* = 947 ms; all *p*s = 1). These outcomes are consistent with the individual condition of Experiment 1.

### Percentages of error trials

As in Experiment 1, the PE results were generally consistent with the RT data. A significant main effect of Previous Trial revealed that PE was lower when the previous trial required the same hand response or when it was a go trial (*M* = 22.76 %) than when the previous trial required a different hand or when it was a no-go trial (*M* = 26.81 %). Task Sequence produced a main effect and interacted with Previous Trial. More importantly, the significant three-way interaction among Task Sequence, Previous Trial, and Task Condition indicated that switch costs depended on the previous trial type, but differently for the full-task and go/no-go conditions. To disentangle this interaction, post hoc tests (Bonferroni corrected) were carried out to examine the effect of Task Sequence separately for the two task conditions.

In the full-task condition, task switch produced a larger PE (*M* = 31.15 %) than did cue repeat (*M* = 26.41 %, *p* = 0.039) and cue switch (*M* = 25.23 %, *p* = 0.046) when the previous trial required a different hand, whereas the latter two did not differ (*p* = 1). Although the results were similar when the previous trial required the same hand, as task switch (*M* = 28.80 %) produced larger PE than did cue repeat (*M* = 22.86 %) or cue switch (*M* = 23.60 %), the differences were only marginal (*p*s = 0.09 and 0.10, respectively). Thus, switch costs were more pronounced when the hand switched across trials than when the same hand repeated. In the go/no-go condition, task switch produced a larger PE (*M* = 27.67 %) than did cue repeat (*M* = 15.30 %, *p* = 0.016) and cue switch (*M* = 15.30 %, *p* = 0.001), whereas the latter two did not differ (*p* = 0.802) when the previous trial was a go trial. When the previous trial was a no-go trial, there were no significant differences (*M*s = 23.45 % for cue repeat, 27.52 % for cue switch, and 27.09 % for task switch; all *p*s > 0.5).

## Discussion

Switch costs were obtained in the go/no-go condition when previous trials were go trials, but not when they were no-go trials, consistent with the individual condition of Experiment 1. In the full-task condition, switch costs were obtained in RT on hand-repeat trials for which participants used the same response set (analogous to actor-repeat trials) as well as on hand-switch trials for which participants used different response sets (analogous to actor-switch trials). The outcomes imply that the same task representation was used to perform trials even when the response set differed between trials as long as the same actor performed both trials. In this experiment, a single actor integrated the two task contexts (i.e., two response sets) into a single task representation that should be equivalent to co-representation of the shared task contexts in the joint condition. Thus, the present results, and their difference with those in the joint condition of Experiment 1, suggest that the lack of switch costs in Experiment 1 was not due to the nature of the task condition (e.g., task complexity, task discrimination, or response set discrimination). Instead, these findings reinforce the conclusion that the lack of switch costs in the joint condition of Experiment 1 implies a lack of task set sharing. Interestingly, there were no cue switch costs on hand-switch trials, indicating that the lack of cue-switch costs in the joint condition of Experiment 1 (which may represent the binding of cues to either one’s own no-reaction or to the other agent and/or his or her response) was not unique to joint performance. It may be that cue-repeat trials still involved switches of the actor- or hand cue, so they were not purely cue-repeat trials as cue encoding continues until the actor- or hand-cue encoding is completed.

## General discussion

Approaches to joint performance agree in assuming that people take the activities of co-actors into account, but there is also evidence that the resulting representations do not capture all aspects of the joint task. The present study sought to test whether people may represent not only the stimuli that co-actors are facing (so as to allow for turn taking; e.g., Wenke et al., 2011) and the actions that co-actors execute (so as to allow for self–other action discrimination; Dolk et al., 2014), but also entire task sets that include stimulus–response relationships relevant only to the co-actor (Knoblich et al., 2011). As we have argued, previous investigations of actor switching (Dudarev & Hassin, 2016; Liefooghe, 2016) are not sufficiently diagnostic because they are open to interpretations that do not require the assumption of task set sharing. A more telling design was discussed by Wenke et al. (2011, Experiment 2), which was taken from an unpublished study. We therefore adopted the basic design from this latter study, together with a manipulation of task cue repetition versus switch to determine whether the basic findings could be confirmed in a well-powered study. Experiment 1 shows that this was indeed the case: task switch costs were obtained after go trials, but not after no-go trials, irrespective of whether a co-actor performed during the actor’s no-go trials. Experiment 2 showed that reliable task switch costs were obtained if the same participant operated on the two response sets that were distributed across two participants in Experiment 1. This ruled out the possibility that the lack of reliable switch costs in Experiment 1 was due to the fact that the two actors used different response sets. Taken together, these findings question a strong claim of task set sharing that “humans represent not only their own tasks but also those of their partners and even those of people who they do not need to coordinate with” (Knoblich et al., 2011, p. 83).

A possible counterargument could be derived from a recent discussion of the processes involved in no-go trials. The lack of switch costs after no-go trials is commonly thought to reflect the fact that task switching is complete only after a response is selected and executed (Philipp, Jolicoeur, Falkenstein, & Koch, 2007; Schuch & Koch, 2003; also see Rogers & Monsell, 1995). On go trials, response-related processes complete the activation of the relevant task set, and the activation survives until the next trial where it can impair the performance if a different task set is required to perform the trial. On no-go trials, however, response-related processes are not executed, so the activation of the relevant task set is not sufficiently strong to carry over to the next trial. This interpretation is consistent with the fact that the elimination of switch costs is associated with increases in RT on repeat trials rather than decreases on switch trials, indicating that repeat trials are performed as if they were switch trials. However, there is an alternative interpretation: the task set may be activated on go and no-go trials alike, but is then suppressed to inhibit responses on no-go trials (Lenartowicz, Yeung, & Cohen, 2011; Verbruggen, Liefooghe, Szmalec, & Vandierendonck, 2005). Consistent with this inhibition account, switch costs are still observed on trials for which only the task cue is presented but no target occurs (cue-only trials; Lenartowicz et al., 2011; Swainson, Martin, & Prosser, 2016). Thus, one could argue that our participants did represent their co-actor’s task, but suppressed the representation on no-go trials. If so, switch costs could not be obtained on trials following no-go trials.

While this would be a valid possibility, the inhibition account faces numerous problems. For one, switch costs are still eliminated when the previous trial presents a neutral stimulus that is not assigned to any response (Astle, Jackson, & Swainson, 2006). No response or task set should be activated under such conditions, so it would not be plausible to inhibit one or the other. Also, it has been shown that switch costs are obtained when one of the alternative responses is inhibited selectively (Verbruggen, Leifooghe, & Vandierendonck, 2006), suggesting that inhibition of a given response is not sufficient to inhibit the entire task set. Most importantly, a task set inhibition account would have predicted negative switch costs in our study, because the account predicts that cuing a co-actor to perform task A in a given no-go trial would lead the actor to activate and then suppress task set A. This should make it particularly difficult for the actor to reactivate the just-suppressed task set A when the task is repeated (Mayr & Keele, 2000; Meuter, & Allport, 1999), which in turn should make repeat trials more demanding than switch trials. Our findings do not provide any support for this prediction. Thus, we conclude that participants did not represent anything more complex or comprehensive than the stimuli that a co-actor is facing and the actions that he or she performs in a joint condition.

Finally, we should note that previous studies used different variations of joint task switching (Dudarev & Hassin, 2016; Liefooghe, 2016) and demonstrated switch costs after the co-actor’s trials. These studies used a condition in which task switching always occurred with switching of the actors, whereas our method separated the two types of switching. It would be important for future studies to examine how the co-occurrences of actor switching and task switching contribute to task-switching costs in joint settings.

## Footnotes

1 We computed post hoc power for the experiments reported by Wenke et al. (2011), assuming a small effect size (Cohen’s *f* = 0.15) with a 2 (Task Transition: repeat vs. switch) × 2 (Condition: joint vs. individual) × 2 (Previous Trial: go vs. no-go) repeated-measures design at the alpha level of 0.05, which resulted in the power of 0.340. With the sample size of 56, the estimated power increased to 0.925, and with the 3 × 2 × 2 design that we actually used in Experiment 1, it went up to 0.979.

2 To implement the Bonferroni correction, *p* values were multiplied by the number of pairwise *t* tests, which is equivalent to dividing the criterion *p* value (*α*) by the number of pairwise *t* tests.
